# Mendelian randomization indicates a causal contribution of type 2 diabetes to retinal vein occlusion

**DOI:** 10.3389/fendo.2023.1146185

**Published:** 2023-05-08

**Authors:** Jian Huang

**Affiliations:** Clinical Laboratory Center, The First Affiliated Hospital of Guangxi Medical University, Nanning, China

**Keywords:** Mendelian randomization, retinal vein occlusion, type 2 diabetes, causal association, risk

## Abstract

**Background:**

Retinal vein occlusion (RVO) is a common retinal vascular disease that can cause severe visual impairment. Many observational studies have shown that type 2 diabetes (T2DM) is associated with RVO, but it remains unknown if the association is causal. The present study aimed to perform Mendelian randomization (MR) analyses to evaluate the causal contribution of genetically predicted T2DM to RVO.

**Methods:**

We obtained summary-level data from a genome-wide association study meta-analysis including 48,286 cases and 250,671 controls for T2DM and from a genome wide association study of 372 cases and 182,573 controls in the FinnGen project for RVO. To verify the robustness of the results, an independent validation dataset for T2DM (12,931 cases and 57,196 controls) was used. In addition to the main MR analysis using the inverse variance weighted (fixed effect) approach, sensitivity analyses and multivariable MR adjusting for common risk factors of RVO were conducted.

**Results:**

Genetically predicted T2DM was found to be causally associated with RVO risk (odds ratio (OR)=2.823, 95% confidence interval (CI): 2.072-3.847, *P*=4.868×10^-11^). This association was supported by sensitivity analyses using the weighted median (OR=2.415, 95% CI: 1.411-4.132, *P*=1.294×10^-3^), weighted mode (OR=2.370, 95% CI: 1.321-4.252, *P*=5.159×10^-3^), maximum likelihood (OR=2.871, 95% CI: 2.100-3.924, *P*=3.719×10^-11^), MR-PRESSO (OR=2.823, 95% CI: 2.135-3.733, *P*=5.150×10^-10^), and MR-Egger (OR=2.441, 95% CI: 1.149-5.184, *P*=2.335×10^-2^) methods. In addition, this association persisted in multivariable MR after accounting for common RVO risk factors (OR=1.748, 95% CI: 1.238-2.467, P=1.490×10^-3^). The MR analyses using the validation dataset obtained consistent results.

**Conclusion:**

This study indicates that genetically predicted T2DM may have a causal contribution to RVO. Future studies are required to elucidate the underlying mechanisms.

## Introduction

Retinal vein occlusion (RVO) is a common retinal vascular disease and an important cause of visual impairment and blindness in all ethnic groups. Depending on location of obstruction, RVO is mainly divided into central retinal vein occlusion (CRVO) and branch retinal vein occlusion (BRVO), with BRVO occurring more commonly than CRVO (1). It is estimated that RVO affects more than 25 million people worldwide (2). The annual incidence of RVO is approximately 15 cases per 100,000 population (3). RVO incidence increases with age, but dose not differ significantly with gender (2). Although exact RVO pathogenesis remains to be elucidated, some risk factors are reported to be associated with RVO, including advanced age, hypertension, and hyperlipidemia (4–9). At current stage, many treatment approaches such as anti-vascular endothelial growth factor agents and corticosteroid therapies are used for RVO, but optimal therapeutic strategies have not yet be established (10).

Type 2 diabetes (T2DM) is a chronic metabolic disease characterized by elevated blood glucose levels due to inadequate insulin secretion (11). Its prevalence has increased rapidly in recent years. According to the findings of a systematic review and meta-analysis by Liu and colleagues (12), the pooled T2DM prevalence was 9.6% (95% confidence interval (CI): 7.3%-12.2%). Epidemiological and clinical studies have noticed that individuals with T2DM have elevated risk of RVO (13–15). For instance, Bhattacharjee et al. (14) recruited 11,182 consecutive T2DM patients by conducting a facility-based opportunistic study to assess the prevalence of RVO. They found that about 3.4% of T2DM patients had RVO. Schwaber and colleagues found a significant relationship between T2DM and RVO (odds ratio (OR)=2.41, 95% CI: 1.68-3.45, *P*<0.001) in an American retrospective case-control study that covered a period of three and one-half years (13). Based on the findings from 37 manuscripts published between 1985 and 2019, Wang and colleagues performed a comprehensive meta-analysis of 148,654 cases and 23,768,820 control subjects to evaluate if DM was a risk factor for RVO (15). Although there was evidence of significant heterogeneity among the collected studies (I^2 ^= 96.6%), they showed that DM was positively associated with elevated RVO risk (OR=1.68, 95% CI: 1.43-1.99).

It should be noted that the majority of research evaluating the association between T2DM and RVO was based on observational studies in which confounding may bias the association. For example, in individuals with T2DM, hyperlipidemia and hypertension are frequently found (11). They might play a role in the observed association between T2DM and RVO. It is necessary to elucidate the causal influence of T2DM on RVO, which will help to better understand how T2DM affects RVO development. Mendelian randomization (MR) can complement observational studies by applying genetic data to examine the causal associations between putative risk factors and a health outcome (16). As far as we know, a MR investigation of the relationship between T2DM and RVO has not been undertaken to date. In this study, we conducted MR analyses to assess the relationship of genetically predicted T2DM with risk of RVO.

## Methods

### Study design and data sources

**Figure 1:**
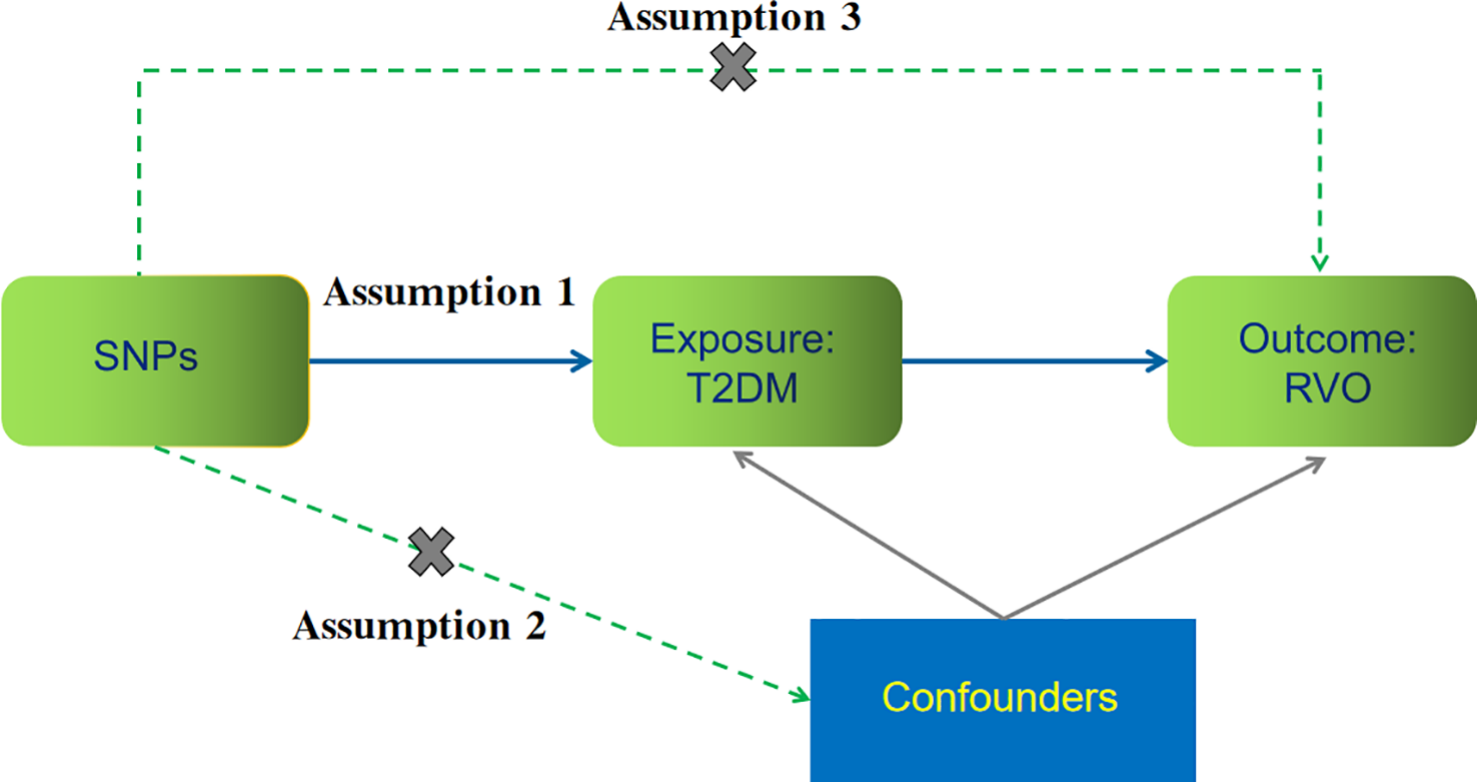
The key assumptions for our Mendelian randomization study evaluating the causal contribution of type 2 diabetes (T2DM) to retinal vein occlusion (RVO). (1) Assumption 1: instrument single nucleotide polymorphisms (SNPs) are strongly associated with T2DM (*P*<5.0×10^−8^); (2) Assumption 2: SNPs are not related to other confounders; and (3) Assumption 3: SNPs affect RVO risk through T2DM but not through other pathways.

We used genome-wide association study (GWAS) summary-level exposure and outcome data from the IEU Open GWAS Project database^1^. Two-sample MR was performed applying summary-level data. **Figure 1** shows the major assumptions of this MR study. We did not seek ethical approval for this MR study because all summary-level data analyzed were publicly available.

### Genetic instruments for T2DM

Summary-level data for single nucleotide polymorphisms (SNPs) associated with T2DM were obtained from a GWAS meta-analysis by Mahajan et al. (17) including 48,286 T2DM cases and 250,671 control subjects of European ancestry. T2DM was diagnosed based on self-reported medical history and International classification of diseases (ICD) codes in linked electronic medical health records (17). We selected T2DM-related SNPs at genome-wide significance (*P*<5.0×10^−8^). For avoiding co-linearity between SNPs, we used the linkage disequilibrium clumping function of the TwosampleMR R package (version 0.5.6)^2^ (18, 19). After linkage disequilibrium clumping (r^2^<0.001) and harmonization of the T2DM summary-level data with the corresponding RVO summary statistics, instrument sets contained 67 instrument SNPs for T2DM. **Supplementary Table 1** shows the details of these instrument SNPs, including rs number, chromosome position, effect allele, other allele, effect size, standard error (SE), and *P* value. For validating the robustness of the causal assessment, we further used another dataset for T2DM from a GWAS analysis by Bonàs-Guarch and colleagues (20), which included 12,931 T2DM cases and 57,196 controls. The characteristics of the instrument SNPs from this dataset are presented in **Supplementary Table 2**.

### RVO outcome data

GWAS summary-level data on RVO were derived from the FinnGen study containing 372 RVO cases and 182,573 controls. The FinnGen study is a national public health project in Finland (21). It seeks to drive genetic insights and better understand disease mechanisms by combining clinical data and genome information. RVO status was diagnosed according to the ICD codes.

### Statistical analyses

The extraction of instrument SNPs, linkage disequilibrium clumping, harmonization of T2DM and RVO summary statistics, and the MR analyses were carried out using the TwosampleMR R package (version 0.5.6) in R (version 4.1.0). The F statistics was used to determine instrument strength; values above ten were considered strong instruments (22). In the main MR analysis evaluating the non-confounded causal association between T2DM and RVO, the inverse variance weighted approach was applied. As described in **Figure 1**, three key assumptions are required for this method (23). To account for potential pleiotropy of the instrument SNPs, sensitivity analyses using weighted median estimator, weighted mode, maximum likelihood, MR-Egger regression, and MR Pleiotropy RESidual Sum and Outlier (MR-PRESSO) approaches were undertaken (24–27). Regarding validity of instruments, each approach makes different assumptions (24–27). The Cochran’s Q test was used to assess between SNP heterogeneity; presence of heterogeneity can reflect pleiotropy of the instrumental variables (28). The *P*-value of the MR-Egger intercept assessment was applied to examine average directional pleiotropy across the instrument SNPs. In addition, a global significance test of the MR-PRESSO method was used for evaluating horizontal pleiotropy. To detect single SNPs that may drive identified causal effects, a leave-one-out analysis using the inverse variance weighted approach was undertaken (29). Furthermore, multivariable MR was conducted in which we adjusted the effect of T2DM on RVO for hypertension, body mass index (BMI), current tobacco smoking, alcohol intake frequency, and levels of high-density lipoprotein cholesterol (HDL-C), low-density lipoprotein cholesterol (LDL-C), and triglycerides. The summary statistics for hypertension (N=462,933), BMI (N=461,460), current tobacco smoking (N=462,434), and alcohol intake frequency (N=462,346) were obtained from the Medical Research Council Integrative Epidemiology Unit (MRC-IEU) consortium, and the summary-level data for HDL-C (N=403,943), LDL-C (N=440,546), and triglycerides (N=441,016) were derived from the UK Biobank. **Table 1** shows the details on these datasets. For multivariable MR, SNPs for each exposure were obtained and combined into a set of all instrument SNPs. After harmonization, the multivariable MR analyses were conducted using the mv_multiple function from the TwosampleMR R package. For all analyses, a two-sided *P*<0.05 was considered statistically significant.

**Table 1:** Details on included GWAS datasets.

Trait	Year	Author or Consortium	Population	GWAS-ID	Sample size
Exposure					
Type 2 diabetes (Discovery)	2018	Mahajan A	European	ebi-a-GCST007515	48,286 cases and 250,671 controls
Type 2 diabetes (Validation)	2018	Bonàs-Guarch S	European	ebi-a-GCST005413	12,931 cases and 57,196 controls
Non-cancer illness code, self-reported: hypertension	2018	MRC-IEU	European	ukb-b-14057	119,731 cases and 343,202 controls
Body mass index	2018	MRC-IEU	European	ukb-b-19953	461,460 participants
Alcohol intake frequency	2018	MRC-IEU	European	ukb-b-5779	462,346 participants
Current tobacco smoking	2018	MRC-IEU	European	ukb-b-223	462,434 participants
HDL cholesterol	2020	UK Biobank	European	ieu-b-109	403,943 participants
LDL cholesterol	2020	UK Biobank	European	ieu-b-110	440,546 participants
Triglycerides	2020	UK Biobank	European	ieu-b-111	441,016 participants
Outcome					
Retinal vein occlusion (central or branch)	2021	FinnGen	European	finn-b-DM_RET_VEIN_OCCLU	372 cases and 182,573 controls

MRC-IEU, Medical Research Council Integrative Epidemiology Unit; GWAS, genome-wide association study; HDL, high-density lipoprotein; LDL, low-density lipoprotein; UK, united kingdom.

## Results

Using the discovery dataset (ebi-a-GCST007515), 67 instrument SNPs were obtained for T2DM after removal of SNPs with linkage disequilibrium and harmonization of the T2DM summary statistics with the corresponding RVO data (**Supplementary Table 1**). The total variance in T2DM explained by these SNPs was 11.15%. The F-statistics of these SNPs was 559.83, indicating that weak instrument bias was unlikely.

**Figure 2:**
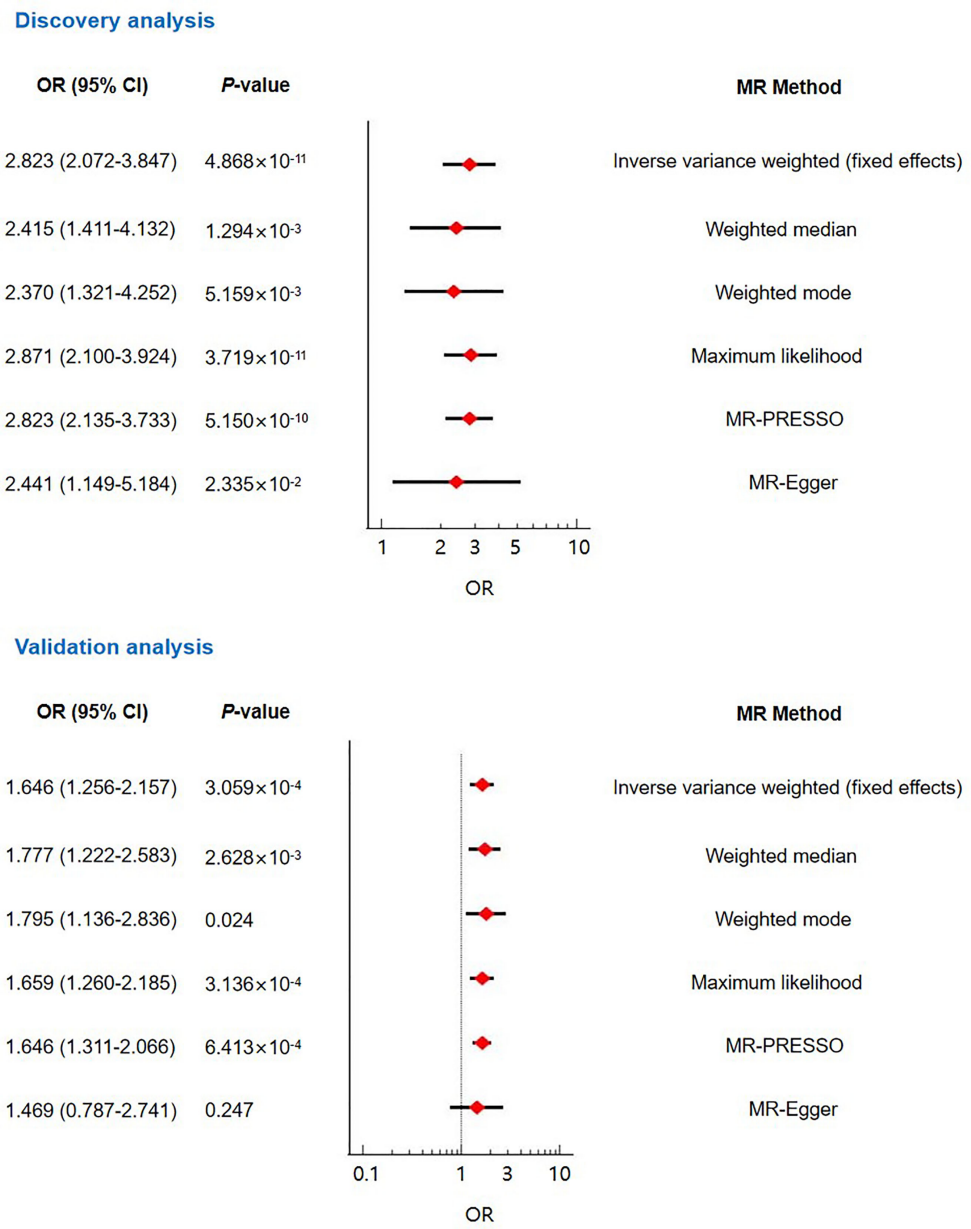
The main Mendelian randomization analyses and sensitivity analyses evaluating the causal contribution of type 2 diabetes to retinal vein occlusion.

We performed the main analysis using the inverse variance weighted (fixed effect) approach. The result suggested that genetically predicted T2DM was associated with enhanced risk of RVO (OR=2.823, 95% CI: 2.072-3.847, *P*=4.868×10^-11^) (**Figure 2** and **Supplementary Figure 1**). This genetically predicted effect was strongly supported by sensitivity analyses using the weighted median (OR=2.415, 95% CI: 1.411-4.132, *P*=1.294×10^-3^), weighted mode (OR=2.370, 95% CI: 1.321-4.252, *P*=5.159×10^-3^), maximum likelihood (OR=2.871, 95% CI: 2.100-3.924, *P*=3.719×10^-11^), MR-PRESSO (OR=2.823, 95% CI: 2.135-3.733, *P*=5.150×10^-10^), and MR-Egger (OR=2.441, 95% CI: 1.149-5.184, *P*=2.335×10^-2^) methods (**Figure 2**). The MR-Egger intercept test indicated the absence of unbalanced directional pleiotropy (intercept: 0.010, *P*=0.679) (**Table 2**). In addition, the MR-PRESSO global test did not detect horizontal pleiotropy (*P*=0.865) (**Table 2**). According to the Cochran’s Q test, there was no evidence of heterogeneity (**Table 2**). No outlier SNPs were identified by the leave-one-out analysis (**Supplementary Table 3**).

**Table 2:** Test for heterogeneity and pleiotropy.

MR analysis	Number of SNPs	Test for heterogeneity		Test for pleiotropy		
		P-value for IVW	P-value for MR-Egger	MR-Egger intercept	Intercept *P*-value	MR-PRESSO global test *P*-value
Discovery analysis	67	0.858	0.841	0.010	0.679	0.865
Validation analysis	16	0.780	0.728	0.021	0.697	0.813

For validating the causal effect of T2DM on RVO, we performed validation analyses using another GWAS dataset (ebi-a-GCST005413) for T2DM. Sixteen instrument SNPs were extracted from this dataset (**Supplementary Table 2**). The F-statistics of these SNPs was 685.05. Both the inverse variance weighted (fixed effect) MR analysis and sensitivity analyses using the weighted median, weighted mode, maximum likelihood, and MR-PRESSO methods suggested causality between T2DM and RVO (**Figure 2** and **Supplementary Figure 2**). No pleiotropy was found according to the MR-Egger intercept test and the MR-PRESSO global test (**Table 2**). The heterogeneity test was not statistically significant (*P*>0.05; **Table 2**). The leave-one-out analysis indicated no excessive influence of a single instrumental variable on the MR estimate (**Supplementary Table 4**).

To further evaluate the robustness of the significant results, we conducted multivariable MR to adjust for the effects of potential confounders including hypertension, BMI, alcohol intake frequency, current tobacco smoking, HDL-C, LDL-C, and triglycerides. Multivariable MR results confirmed that genetically predicted T2DM was significantly associated with increased RVO risk (**Table 3**).

**Table 3:** Multivariable MR on the causal association between T2DM and RVO.

Adjustment for confounding factors	T2DM dataset	Number of SNPs	RVO dataset	OR	95% CI	*P*-value
Hypertension, BMI, alcohol intake frequency, current tobacco smoking, HDL cholesterol, LDL cholesterol, and triglycerides	ebi-a-GCST007515 (Discovery)	30	finn-b-DM_RET_VEIN_OCCLU	1.748	1.238-2.467	1.490×10^-3^
Hypertension, BMI, alcohol intake frequency, current tobacco smoking, HDL cholesterol, LDL cholesterol, and triglycerides	ebi-a-GCST005413 (Validation)	5	finn-b-DM_RET_VEIN_OCCLU	1.433	1.132-1.814	2.783×10^-3^

BMI, body mass index; OR, odds ratio; CI, confidence interval; T2DM, type 2 diabetes; RVO, retinal vein occlusion; HDL, high-density lipoprotein; LDL, low-density lipoprotein.

## Discussion

The application of summary-level statistics in MR studies has become an increasingly popular approach for causal inference and provided new insights into elucidating disease pathogenesis. This MR study aimed to evaluate a causal relationship between T2DM and RVO using summary-level data from European participants. Our results showed that genetically predicted T2DM was positively associated with a greater risk of developing RVO. To our knowledge, this is the first MR study providing evidence that T2DM may be an important contributor to the development of RVO.

To date, studies using traditional observational methods have reported inconsistent findings regarding the association between T2DM and RVO. For example, An Indian facility-based opportunistic investigation evaluating the prevalence of RVO among T2DM patients reported that 3.4% of T2DM patients developed RVO (14). In a retrospective, matched cohort study, Chang et al. (30) recruited 240,761 individuals with DM and 240,761 subjects without DM from 2003 to 2005 for investigating whether DM contributed to a higher risk of RVO; age, sex, and index date were matched for the two groups. The authors found that compared with the control group, individuals with DM had an increased risk for RVO (incidence rate ratio=1.91, 95% CI: 1.75-2.08). Similarly, Schwaber et al. (13) reported that there was a dramatically elevated risk of RVO among patients with T2DM in comparison to age-matched control subjects (OR=2.41, 95% CI: 1.68-3.45, *P*<0.001). However, some studies did not observe any association between T2DM and RVO. Mitchell et al. (31) performed a population-based survey in a representative Australian population sample (Blue Mountains Eye Study) to assess the prevalence of RVO and its associated risk factors. The authors reported a prevalence of 1.6% (CI: 1.3-1.9) among both male and female residents, but they found no association of DM with RVO in a logistic regression analysis controlling for age and glaucoma (OR=1.4, 95%: 0.5-3.5). In a cross-sectional study involving 1775 Japanese participants aged 40 years or older, Yasuda et al. (32) found that DM was not associated with RVO risk after adjusting for age and sex (OR=0.65, 95% CI: 0.23-1.87). Between these studies, we noticed that remarkable heterogeneity was present in race, study design, number of participants, participant characteristics, ascertainment of RVO, RVO prevalence, and statistical methodologies, which may account for the inconsistent findings. As important risk factors for RVO, hypertension and hyperlipidemia were not routinely adjusted for in the epidemiological data.

Our univariable MR analysis using the inverse variance weighted method suggested a significant causal association between T2DM and RVO. This association was confirmed by sensitivity analyses. However, only univariable analysis could not reveal the direct effect of T2DM on RVO. It was thus necessary to evaluate if T2DM was independently associated with RVO by conducting summary data multivariable MR. Like the univariable MR, we used a validation dataset for T2DM in the multivariable MR. Although ORs attenuated after adjusting for the confounders, the significant causal association remained in the multivariable MR, indicating that T2DM was independently associated with RVO.

Our findings supported those from the studies by Schwaber et al. (13), Bhattacharjee et al. (14), and Chang et al. (30), and were consistent with three previous comprehensive meta-analysis (15, 33, 34). O’Mahoney et al. (33) collected relevant studies published between 1985 and 2007 and meta-analyzed them for evaluating the relationship between RVO and traditional risk factors of atherosclerosis. The authors observed a significant association of DM with any form of RVO (pooled OR=1.5, 95% CI: 1.1-2.0). A recent large-scale meta-analysis by Wang et al. (15) for DM and RVO identified 37 publications from 38 studies published between 1985 and 2019 with a total of 148,654 RVO cases and 23,768,820 controls. Wang et al. (15) revealed that DM was significantly associated with risk of any type of RVO (OR=1.68, 95% CI: 1.43-1.99). In addition, they demonstrated the positive association in different populations, including Italians (OR = 2.16, 95% CI: 1.22-3.83), Americans (OR=1.4, 95% CI: 1.01-1.94), and Turks (OR = 2.09, 95% CI: 1.48-2.93). Similar findings were also reported in a meta-analysis by Kolar (34). It is worth mentioning that relationships assessed in meta-analyses summarizing conventional observational studies are not equivalent to those evaluated using MR estimates. Because MR takes into account relationships of life-time and cumulative genetic risk. Compared with the previous meta-analyses, our study has several merits. Firstly, the MR design enabled us to evaluate a causal contribution of T2DM to RVO, but previous studies could not. Secondly, the application of univariable and multivariable MR analyses and a series of sensitivity analyses made our results more resistant to confounding. Thirdly, the use of a replication dataset for T2DM enhanced the reliability of our results.

T2DM contributes to retinal vascular endothelial cell dysfunction and significantly affects the microvascular circulation in the retina (35–37). It can disrupt pericytes-endothelial interactions, damage endothelial cells and the basement membrane, and induce structural alteration of retinal vessel walls, leading to retinal microvascular rigidity and abnormal blood flow (36, 38). These changes may facilitate the formation of thrombus and downstream venous occlusion. Additionally, it is well acknowledged that T2DM is associated with oxidative stress and inflammation which are important contributors to RVO development (39). Oxidative stress can induce mitochondrial DNA damage, disrupt the functioning of multiple vascular cells, promote endothelial cell apoptosis, and increase blood viscosity, playing a prominent role in retinal vascular impairment and RVO pathogenesis (40–42). Increased levels of inflammatory mediators including soluble intercellular adhesion molecule 1, vascular endothelial growth factor 1, monocyte chemotactic protein 1, and interleukin 6 were observed in both T2DM and RVO patients (43–46). These factors might mediate the effect of T2DM on RVO. The underlying molecular mechanisms by which T2DM promotes RVO development remains poorly understood. It is a topic deserving of further research.

Some potential limitation of our study should be mentioned. Firstly, Both T2DM and RVO summary statistics were obtained from individuals of European ancestry. This reduced population structure bias, but limited the generalizability of our finding to other ethnicities. In the Hisayama study performed in a general Japanese population, Yasuda et al. (32) observed a higher prevalence of RVO (2.1%) than that reported in European populations. In addition, they found that DM was not related to RVO. A causal relationship between genetically predicted T2DM and RVO should be further evaluated in Asians when relevant data are available. Secondly, we did not evaluate the association of genetically predicted T2DM with CRVO and BRVO respectively, because such information was unavailable. Thirdly, although common risk factors for RVO were adjusted for in our analyses, we could not completely rule out the effects of other unexplored factors.

## Conclusion

In summary, our MR study showed that genetically predicted T2DM was causally related to RVO in individuals of European ancestry. This finding suggested that prevention and control of T2DM may be beneficial for preventing RVO in European populations.

## Data availability statement

We analyzed publicly available datasets which can be found here: The IEU Open GWAS Project database (https://gwas.mrcieu.ac.uk/).

## Ethics statement

This mendelian randomization study only analyzed publicly available summary statistics, so it was exempt from ethical approval.

## Author contributions

JH designed the study, collected data, performed statistical analyses, and wrote the manuscript. The author contributed to the article and approved the submitted version.

## Footnotes

1
https://gwas.mrcieu.ac.uk/.

2
https://mrcieu.github.io/TwoSampleMR/index.html.

## Supplementary material

Supplementary Figure 1 Scatter plot representing the significant causal contribution of genetically predicted type 2 diabetes (discovery dataset; ebi-a-GCST007515) to retinal vein occlusion.

Supplementary Figure 2 Scatter plot representing the significant causal contribution of genetically predicted type 2 diabetes (validation dataset; ebi-a-GCST005413) to retinal vein occlusion.
