# Supplementary figures and images for: Mendelian randomization indicates a causal contribution of type 2 diabetes to retinal vein occlusion
Source: Front Endocrinol (Lausanne). 2023 May 8;14:1146185. doi: 10.3389/fendo.2023.1146185 (PMC10200935; doi:10.3389/fendo.2023.1146185)

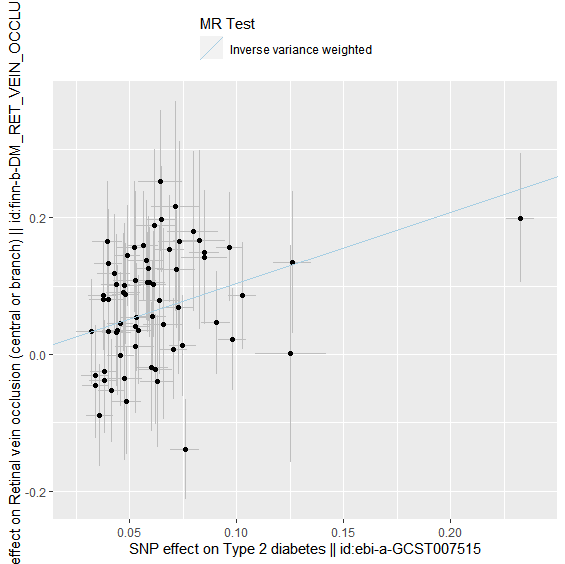

Supplement: Supplementary Figure 1 — Scatter plot representing the significant causal contribution of genetically predicted type 2 diabetes (discovery dataset; ebi-a-GCST007515) to retinal vein occlusion. [file Image_1.tif]

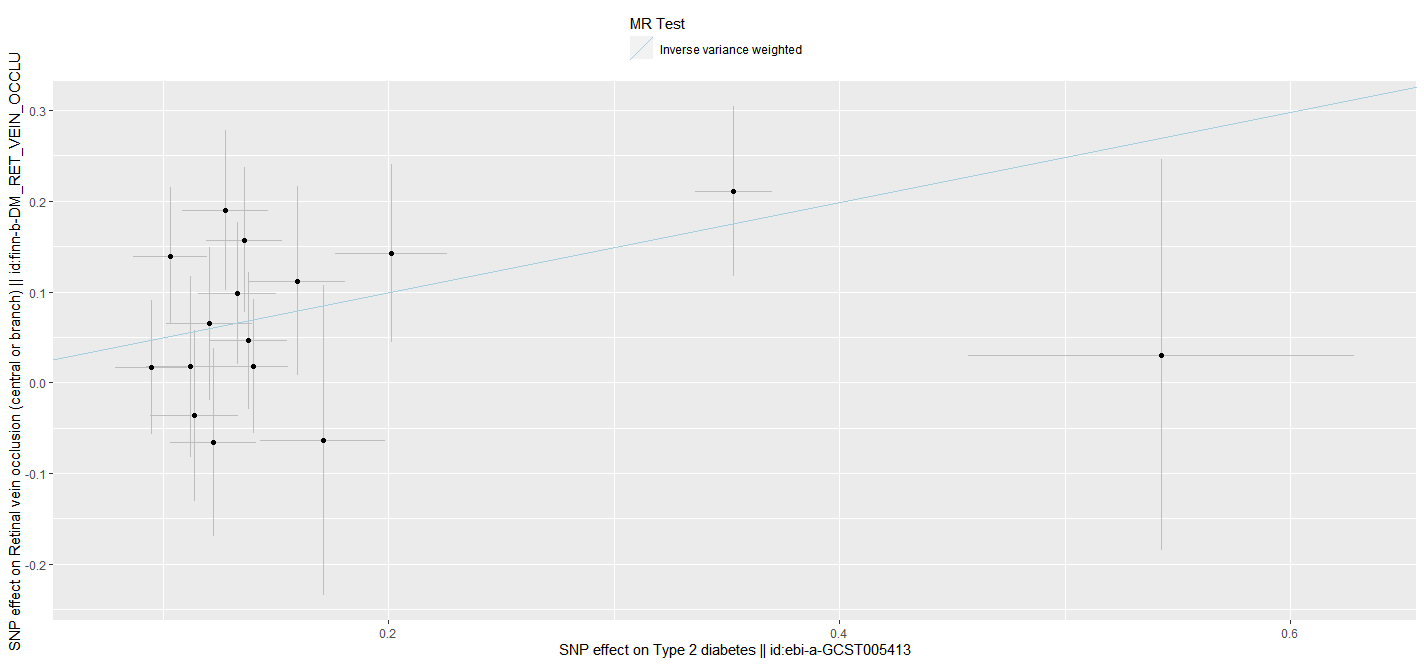

Supplement: Supplementary Figure 2 — Scatter plot representing the significant causal contribution of genetically predicted type 2 diabetes (validation dataset; ebi-a-GCST005413) to retinal vein occlusion. [file Image_2.tif]
