# Supplementary material for: Mendelian randomization indicates a causal contribution of type 2 diabetes to retinal vein occlusion
Source: Front Endocrinol (Lausanne). 2023 May 8;14:1146185. doi: 10.3389/fendo.2023.1146185 (PMC10200935; doi:10.3389/fendo.2023.1146185)
Supplement: Supplementary file 3 [file Table_1.docx]

Supplementary Table 1 Characteristics of the SNPs used as instruments for T2DM from the dataset of ebi-a-GCST007515.

| SNP | Chr | Position | EA | NEA | EAF | Beta | SE | *P* |
| --- | --- | --- | --- | --- | --- | --- | --- | --- |
| rs10146997 | 14 | 79945162 | G | A | 0.225 | 0.047 | 0.008 | 1.84E-10 |
| rs10758593 | 9 | 4292083 | A | G | 0.424 | 0.048 | 0.006 | 1.95E-16 |
| rs1077394 | 6 | 31610384 | T | C | 0.651 | 0.040 | 0.007 | 7.64E-10 |
| rs10830963 | 11 | 92708710 | G | C | 0.285 | 0.085 | 0.007 | 1.35E-32 |
| rs10842994 | 12 | 27965150 | T | C | 0.178 | -0.063 | 0.008 | 9.43E-16 |
| rs10906115 | 10 | 12314997 | G | A | 0.397 | -0.034 | 0.006 | 1.28E-08 |
| rs10965250 | 9 | 22133284 | A | G | 0.180 | -0.126 | 0.009 | 9.73E-47 |
| rs11603334 | 11 | 72432985 | A | G | 0.149 | -0.072 | 0.009 | 9.50E-18 |
| rs11708067 | 3 | 123065778 | G | A | 0.206 | -0.073 | 0.008 | 3.56E-24 |
| rs12571751 | 10 | 80942631 | G | A | 0.461 | -0.054 | 0.006 | 3.39E-21 |
| rs12602912 | 17 | 65870073 | T | C | 0.238 | 0.043 | 0.008 | 4.74E-09 |
| rs1260326 | 2 | 27730940 | C | T | 0.630 | 0.061 | 0.006 | 5.31E-25 |
| rs13266634 | 8 | 118184783 | T | C | 0.309 | -0.090 | 0.007 | 1.85E-47 |
| rs13389219 | 2 | 165528876 | T | C | 0.378 | -0.065 | 0.007 | 2.75E-24 |
| rs1359790 | 13 | 80717156 | A | G | 0.268 | -0.059 | 0.007 | 2.90E-17 |
| rs1531343 | 12 | 66174894 | C | G | 0.126 | 0.066 | 0.010 | 1.31E-13 |
| rs1558902 | 16 | 53803574 | A | T | 0.373 | 0.098 | 0.007 | 2.45E-47 |
| rs17782313 | 18 | 57851097 | C | T | 0.246 | 0.044 | 0.007 | 3.50E-10 |
| rs1801212 | 4 | 6302519 | A | G | 0.748 | 0.068 | 0.007 | 1.10E-24 |
| rs1801282 | 3 | 12393125 | G | C | 0.113 | -0.085 | 0.011 | 1.36E-17 |
| rs2191349 | 7 | 15064309 | T | G | 0.550 | 0.049 | 0.006 | 1.17E-18 |
| rs2206277 | 6 | 50798526 | T | C | 0.195 | 0.047 | 0.008 | 4.31E-10 |
| rs2237895 | 11 | 2857194 | C | A | 0.407 | 0.076 | 0.007 | 1.96E-29 |
| rs2296172 | 1 | 39835817 | G | A | 0.193 | 0.060 | 0.008 | 6.73E-16 |
| rs2307111 | 5 | 75003678 | C | T | 0.438 | -0.053 | 0.007 | 1.57E-15 |
| rs2395163 | 6 | 32387809 | C | T | 0.200 | 0.052 | 0.008 | 1.34E-11 |
| rs243021 | 2 | 60584819 | A | G | 0.479 | 0.046 | 0.006 | 4.65E-14 |
| rs2796441 | 9 | 84308948 | A | G | 0.411 | -0.038 | 0.006 | 1.08E-09 |
| rs2925979 | 16 | 81534790 | C | T | 0.695 | -0.038 | 0.007 | 2.49E-08 |
| rs2943641 | 2 | 227093745 | C | T | 0.672 | 0.059 | 0.007 | 1.87E-18 |
| rs328 | 8 | 19819724 | G | C | 0.097 | -0.052 | 0.010 | 6.82E-09 |
| rs340874 | 1 | 214159256 | C | T | 0.503 | 0.045 | 0.006 | 1.41E-14 |
| rs35658696 | 5 | 102338811 | G | A | 0.045 | 0.125 | 0.017 | 1.18E-16 |
| rs35720761 | 2 | 43519977 | T | C | 0.105 | -0.073 | 0.010 | 4.58E-15 |
| rs3764002 | 12 | 108618630 | T | C | 0.282 | -0.032 | 0.007 | 3.33E-08 |
| rs41278853 | 22 | 30416527 | G | A | 0.074 | -0.083 | 0.012 | 5.61E-13 |
| rs4457053 | 5 | 76424949 | A | G | 0.730 | -0.040 | 0.007 | 8.81E-11 |
| rs4502156 | 15 | 62383155 | C | T | 0.476 | -0.036 | 0.006 | 1.20E-09 |
| rs459193 | 5 | 55806751 | G | A | 0.699 | 0.053 | 0.007 | 2.82E-15 |
| rs4607103 | 3 | 64711904 | T | C | 0.265 | -0.040 | 0.007 | 9.35E-09 |
| rs4812831 | 20 | 43018260 | A | G | 0.131 | 0.062 | 0.010 | 8.48E-10 |
| rs5015480 | 10 | 94465559 | T | C | 0.458 | -0.071 | 0.006 | 1.57E-30 |
| rs505922 | 9 | 136149229 | C | T | 0.371 | 0.038 | 0.007 | 9.07E-10 |
| rs516946 | 8 | 41519248 | C | T | 0.779 | 0.066 | 0.007 | 1.87E-20 |
| rs5219 | 11 | 17409572 | C | T | 0.636 | -0.058 | 0.006 | 5.68E-22 |
| rs55834942 | 12 | 121437114 | A | G | 0.170 | -0.058 | 0.008 | 5.45E-13 |
| rs58542926 | 19 | 19379549 | T | C | 0.076 | 0.072 | 0.011 | 4.77E-12 |
| rs60980157 | 9 | 139235415 | T | C | 0.229 | -0.062 | 0.008 | 3.19E-16 |
| rs6813195 | 4 | 153520475 | T | C | 0.310 | -0.049 | 0.007 | 1.10E-13 |
| rs7177055 | 15 | 77832762 | A | G | 0.658 | 0.054 | 0.007 | 7.54E-15 |
| rs7202877 | 16 | 75247245 | G | T | 0.111 | -0.064 | 0.010 | 5.63E-12 |
| rs730497 | 7 | 44223721 | A | G | 0.162 | 0.048 | 0.008 | 4.01E-10 |
| rs731839 | 19 | 33899065 | A | G | 0.635 | -0.038 | 0.007 | 5.15E-10 |
| rs738409 | 22 | 44324727 | G | C | 0.239 | 0.040 | 0.007 | 2.12E-10 |
| rs7501939 | 17 | 36101156 | C | T | 0.622 | -0.061 | 0.006 | 3.40E-24 |
| rs7572857 | 2 | 65296798 | A | G | 0.154 | -0.052 | 0.009 | 8.25E-09 |
| rs7633675 | 3 | 185510613 | G | T | 0.348 | 0.097 | 0.006 | 4.26E-55 |
| rs769449 | 19 | 45410002 | A | G | 0.113 | -0.064 | 0.010 | 7.56E-13 |
| rs7756992 | 6 | 20679709 | G | A | 0.316 | 0.103 | 0.007 | 1.86E-59 |
| rs781831 | 17 | 3947644 | C | T | 0.422 | 0.041 | 0.006 | 8.26E-11 |
| rs7903146 | 10 | 114758349 | T | C | 0.267 | 0.233 | 0.007 | 1.00E-200 |
| rs8042680 | 15 | 91521337 | A | C | 0.434 | 0.044 | 0.007 | 2.32E-11 |
| rs8108269 | 19 | 46158513 | G | T | 0.326 | 0.056 | 0.007 | 8.26E-17 |
| rs864745 | 7 | 28180556 | C | T | 0.457 | -0.075 | 0.006 | 1.17E-34 |
| rs9379084 | 6 | 7231843 | A | G | 0.116 | -0.080 | 0.012 | 1.14E-13 |
| rs9388489 | 6 | 126698719 | G | A | 0.511 | 0.034 | 0.007 | 1.60E-08 |
| rs972283 | 7 | 130466854 | G | A | 0.560 | 0.044 | 0.007 | 1.66E-11 |

T2DM, type 2 diabetes; SNP, single nucleotide polymorphism; EA, effect allele; EAF, effect allele frequency; NEA, non-effect allele; SE, standard error.
