# Supplementary material for: Mendelian randomization indicates a causal contribution of type 2 diabetes to retinal vein occlusion
Source: Front Endocrinol (Lausanne). 2023 May 8;14:1146185. doi: 10.3389/fendo.2023.1146185 (PMC10200935; doi:10.3389/fendo.2023.1146185)
Supplement: Supplementary file 4 [file Table_2.docx]

Supplementary Table 2 Characteristics of the SNPs used as instruments for T2DM from the dataset of ebi-a-GCST005413.

| SNP | Chr | Position | EA | NEA | EAF | Beta | SE | *P* |
| --- | --- | --- | --- | --- | --- | --- | --- | --- |
| rs10811662 | 9 | 22134253 | A | G | 0.171 | -0.160 | 0.021 | 1.00E-13 |
| rs11257655 | 10 | 12307894 | T | C | 0.210 | 0.120 | 0.019 | 3.74E-10 |
| rs13266634 | 8 | 118184783 | T | C | 0.305 | -0.138 | 0.017 | 3.01E-15 |
| rs1708302 | 7 | 28198677 | T | C | 0.492 | -0.140 | 0.016 | 2.93E-19 |
| rs2943656 | 2 | 227121918 | G | A | 0.634 | 0.103 | 0.016 | 2.74E-10 |
| rs34872471 | 10 | 114754071 | C | T | 0.701 | 0.353 | 0.017 | 1.15E-94 |
| rs35261542 | 6 | 20675792 | A | C | 0.277 | 0.133 | 0.017 | 1.66E-14 |
| rs3768321 | 1 | 40035928 | T | G | 0.197 | 0.112 | 0.020 | 9.02E-09 |
| rs3843467 | 5 | 55856375 | T | G | 0.203 | 0.122 | 0.019 | 1.90E-10 |
| rs4746890 | 10 | 71470390 | C | T | 0.783 | -0.114 | 0.019 | 4.41E-09 |
| rs6743071 | 2 | 43586287 | G | T | 0.904 | -0.171 | 0.028 | 5.77E-10 |
| rs71304101 | 3 | 12396913 | A | G | 0.122 | -0.201 | 0.025 | 4.39E-16 |
| rs71320321 | 3 | 185519107 | A | G | 0.318 | 0.136 | 0.017 | 5.05E-16 |
| rs76895963 | 12 | 4384844 | G | T | 0.979 | -0.543 | 0.086 | 2.27E-10 |
| rs7903302 | 10 | 94429511 | C | T | 0.576 | -0.095 | 0.016 | 3.52E-09 |
| rs9268835 | 6 | 32428115 | A | G | 0.291 | 0.128 | 0.019 | 1.37E-11 |

T2DM, type 2 diabetes; SNP, single nucleotide polymorphism; EA, effect allele; NEA, non-effect allele; EAF, effect allele frequency; SE, standard error.
