# Supplementary material for: Mendelian randomization indicates a causal contribution of type 2 diabetes to retinal vein occlusion
Source: Front Endocrinol (Lausanne). 2023 May 8;14:1146185. doi: 10.3389/fendo.2023.1146185 (PMC10200935; doi:10.3389/fendo.2023.1146185)
Supplement: Supplementary file 5 [file Table_3.docx]

Supplementary Table 3 Leave-one-out analysis using the dataset of ebi-a-GCST007515.

|  | SNP | OR | 95% lower confidence interval | 95% upper confidence interval |
| --- | --- | --- | --- | --- |
|  | All | 2.823 | 2.072 | 3.847 |
| Removing | rs10146997 | 2.805 | 2.056 | 3.826 |
| Removing | rs10758593 | 2.800 | 2.051 | 3.821 |
| Removing | rs1077394 | 2.783 | 2.040 | 3.796 |
| Removing | rs10830963 | 2.761 | 2.016 | 3.780 |
| Removing | rs10842994 | 2.874 | 2.106 | 3.923 |
| Removing | rs10906115 | 2.856 | 2.095 | 3.895 |
| Removing | rs10965250 | 2.820 | 2.058 | 3.865 |
| Removing | rs11603334 | 2.789 | 2.041 | 3.810 |
| Removing | rs11708067 | 2.827 | 2.070 | 3.861 |
| Removing | rs12571751 | 2.838 | 2.078 | 3.875 |
| Removing | rs12602912 | 2.794 | 2.048 | 3.810 |
| Removing | rs1260326 | 2.795 | 2.046 | 3.818 |
| Removing | rs13266634 | 2.879 | 2.101 | 3.945 |
| Removing | rs13389219 | 2.724 | 1.994 | 3.722 |
| Removing | rs1359790 | 2.795 | 2.047 | 3.817 |
| Removing | rs1531343 | 2.829 | 2.074 | 3.858 |
| Removing | rs1558902 | 2.929 | 2.135 | 4.018 |
| Removing | rs17782313 | 2.827 | 2.073 | 3.856 |
| Removing | rs1801212 | 2.757 | 2.018 | 3.769 |
| Removing | rs1801282 | 2.789 | 2.041 | 3.812 |
| Removing | rs2191349 | 2.765 | 2.026 | 3.774 |
| Removing | rs2206277 | 2.803 | 2.055 | 3.823 |
| Removing | rs2237895 | 3.054 | 2.232 | 4.179 |
| Removing | rs2296172 | 2.863 | 2.098 | 3.907 |
| Removing | rs2307111 | 2.833 | 2.075 | 3.868 |
| Removing | rs2395163 | 2.782 | 2.040 | 3.796 |
| Removing | rs243021 | 2.852 | 2.090 | 3.893 |
| Removing | rs2796441 | 2.855 | 2.093 | 3.894 |
| Removing | rs2925979 | 2.803 | 2.055 | 3.823 |
| Removing | rs2943641 | 2.778 | 2.034 | 3.794 |
| Removing | rs328 | 2.811 | 2.062 | 3.833 |
| Removing | rs340874 | 2.825 | 2.070 | 3.855 |
| Removing | rs35658696 | 2.870 | 2.101 | 3.920 |
| Removing | rs35720761 | 2.802 | 2.054 | 3.822 |
| Removing | rs3764002 | 2.823 | 2.071 | 3.850 |
| Removing | rs41278853 | 2.796 | 2.049 | 3.816 |
| Removing | rs4457053 | 2.809 | 2.060 | 3.831 |
| Removing | rs4502156 | 2.882 | 2.113 | 3.931 |
| Removing | rs459193 | 2.824 | 2.069 | 3.855 |
| Removing | rs4607103 | 2.827 | 2.073 | 3.855 |
| Removing | rs4812831 | 2.779 | 2.037 | 3.792 |
| Removing | rs5015480 | 2.885 | 2.110 | 3.945 |
| Removing | rs505922 | 2.803 | 2.055 | 3.824 |
| Removing | rs516946 | 2.837 | 2.078 | 3.874 |
| Removing | rs5219 | 2.789 | 2.042 | 3.810 |
| Removing | rs55834942 | 2.782 | 2.038 | 3.798 |
| Removing | rs58542926 | 2.793 | 2.048 | 3.809 |
| Removing | rs60980157 | 2.883 | 2.111 | 3.937 |
| Removing | rs6813195 | 2.891 | 2.119 | 3.946 |
| Removing | rs7177055 | 2.836 | 2.077 | 3.871 |
| Removing | rs7202877 | 2.819 | 2.066 | 3.846 |
| Removing | rs730497 | 2.843 | 2.085 | 3.876 |
| Removing | rs731839 | 2.858 | 2.095 | 3.898 |
| Removing | rs738409 | 2.778 | 2.037 | 3.789 |
| Removing | rs7501939 | 2.828 | 2.071 | 3.862 |
| Removing | rs7572857 | 2.840 | 2.082 | 3.874 |
| Removing | rs7633675 | 2.761 | 2.015 | 3.784 |
| Removing | rs769449 | 2.743 | 2.010 | 3.744 |
| Removing | rs7756992 | 2.848 | 2.076 | 3.908 |
| Removing | rs781831 | 2.874 | 2.107 | 3.921 |
| Removing | rs7903146 | 2.916 | 2.084 | 4.081 |
| Removing | rs8042680 | 2.830 | 2.075 | 3.861 |
| Removing | rs8108269 | 2.760 | 2.022 | 3.769 |
| Removing | rs864745 | 2.888 | 2.111 | 3.952 |
| Removing | rs9379084 | 2.783 | 2.039 | 3.799 |
| Removing | rs9388489 | 2.853 | 2.092 | 3.890 |
| Removing | rs972283 | 2.791 | 2.046 | 3.808 |

SNP, single nucleotide polymorphism; OR, odds ratio.
