# Supplementary material for: Mendelian randomization indicates a causal contribution of type 2 diabetes to retinal vein occlusion
Source: Front Endocrinol (Lausanne). 2023 May 8;14:1146185. doi: 10.3389/fendo.2023.1146185 (PMC10200935; doi:10.3389/fendo.2023.1146185)
Supplement: Supplementary file 6 [file Table_4.docx]

Supplementary Table 4 Leave-one-out analysis using the dataset of ebi-a-GCST005413.

|  | SNP | OR | 95% lower confidence interval | 95% upper confidence interval |
| --- | --- | --- | --- | --- |
|  | All | 1.646 | 1.256 | 2.157 |
| Removing | rs10811662 | 1.630 | 1.236 | 2.150 |
| Removing | rs11257655 | 1.643 | 1.247 | 2.165 |
| Removing | rs13266634 | 1.664 | 1.258 | 2.201 |
| Removing | rs1708302 | 1.691 | 1.278 | 2.238 |
| Removing | rs2943656 | 1.595 | 1.211 | 2.101 |
| Removing | rs34872471 | 1.587 | 1.157 | 2.178 |
| Removing | rs35261542 | 1.623 | 1.229 | 2.144 |
| Removing | rs3768321 | 1.660 | 1.262 | 2.183 |
| Removing | rs3843467 | 1.693 | 1.287 | 2.227 |
| Removing | rs4746890 | 1.685 | 1.281 | 2.217 |
| Removing | rs6743071 | 1.674 | 1.274 | 2.200 |
| Removing | rs71304101 | 1.616 | 1.219 | 2.143 |
| Removing | rs71320321 | 1.584 | 1.199 | 2.092 |
| Removing | rs76895963 | 1.748 | 1.310 | 2.333 |
| Removing | rs7903302 | 1.663 | 1.264 | 2.190 |
| Removing | rs9268835 | 1.580 | 1.199 | 2.082 |

SNP, single nucleotide polymorphism; OR, odds ratio.
